# A versatile conformal circularly polarized quad-element antenna for X-band applications

**DOI:** 10.1038/s41598-024-79197-2

**Published:** 2024-11-12

**Authors:** P. Sundaravadivel, D. Rajesh Kumar, Yuvaraj Padmanaban, Om Prakash Kumar

**Affiliations:** 1 Saveetha Engineering College, Chennai, India; 2 Vel Tech Rangarajan Dr. Sagunthala R&D Institute of Science and Technology, Chennai, India; 3 Department of Electronics and Communication Engineering, Manipal Institute of Technology, Manipal Academy of Higher Education, Manipal, 576104 India

**Keywords:** Conformal, X-band, Circular polarization, Axial ratio, MIMO, Engineering, Aerospace engineering, Electrical and electronic engineering

## Abstract

This article presents a flexible four-element antenna for X-band applications. The proposed antenna covers the spectrum ranging from 10.6 to 11.9 GHz, including a significant portion of the X-band. The single antenna element comprises a modified E-shaped radiating patch, which is fed by a unique feeding structure consisting of a 50-Ω feeding strip connected with two rectangular stubs on both sides. All antenna elements are printed on a flexible felt 60 mm × 60 mm × 1 mm (2.12 λ × 2.12 λ × 0.035 λ at 10.6 GHz) substrate. The proposed antenna exhibits circular polarization over the desired bans of operation with an axial ratio (< 3 dB) bandwidth of 1.2 GHz, which is significant for X-band applications. The designed antenna is manufactured and practically tested. Both simulated and measured results agree well with each other, ensuring the optimal performance of the antenna. Moreover, the antenna achieves a maximum gain of up to 8 dBi in the desired band of operation. Further MIMO parameters, including envelope correlation coefficient (ECC), diversity gain (DG), channel capacity loss (CCL), and mean effective gain (MEG), are calculated to validate the MIMO performance. Finally, a conformal analysis was carried out to study the robustness of the antenna in various bending scenarios. Results show that the antenna reported in this article is the correct choice for conformal X-band MIMO applications.

## Introduction

MIMO technology enhances wireless communication with array configurations and multiple antennas, improving data speed and reliability^1^. It boosts spectral efficiency and reduces channel fading and interference effects, vital for modern wireless systems. Yet, improving isolation in compact MIMO systems remains challenging^2^. As the number of antennas increases in a confined space, interference and crosstalk risk rise, potentially degrading system performance. Ensuring sufficient isolation between antennas is crucial to prevent these effects and ensure reliable communication. Innovative design approaches, including optimized placement, advanced isolation techniques, and integrated shielding mechanisms, are necessary while maintaining device compactness. Overcoming this challenge is vital to unleashing MIMO’s full potential and delivering robust wireless solutions. The literature has introduced numerous MIMO antennas with diverse designs and isolation methods^3–8^. These studies highlight researchers’ ingenuity in addressing isolation challenges in MIMO systems. Some methods prioritize physical antenna separation, while others utilize inventive antenna structures or integrate isolation components into designs. Techniques like polarization diversity, beamforming, frequency diversity, and adaptive signal processing are explored to reduce interference and boost MIMO system performance. Through evaluation and comparison, researchers seek optimal strategies for high-performance MIMO antennas meeting modern wireless communication demands.

In Refs.^3,4^, isolation enhancement involves positioning MIMO antenna elements orthogonally, exploiting spatial diversity to reduce interference and enhance signal reliability. Reference^4^ additionally embeds a parasitic structure between elements, manipulating electromagnetic fields to attenuate unwanted coupling and improve isolation. This combination of orthogonal placement and parasitic structures demonstrates an innovative approach to interference mitigation and MIMO antenna system robustness. In Refs.^5–8^, four-element MIMO antennas are introduced, advancing MIMO antenna design. Reference^5^ describes an antenna with a meander dipole, reflector, and parasitic strip. The design positions four antenna elements orthogonally in a square loop configuration, reducing antenna size and enhancing isolation. By leveraging this design, researchers aim for compact yet high-performance MIMO antennas suitable for diverse wireless applications. The antenna achieves an impressive impedance bandwidth (IBW) of 23.9%, from 0.63 to 2.95 GHz. Isolations between adjacent elements are less than − 14 dB, while they reach − 18 dB between opposite elements, effectively suppressing interference and crosstalk. These levels enhance performance and reliability in MIMO systems. Despite its capabilities, the antenna maintains a compact 85 × 85 mm^2^ form factor. This antenna’s wide bandwidth, high isolation, and compact size suit a variety of wireless communication applications where space and performance are critical. The envelope correlation coefficient (ECC) between adjacent elements is less than 0.008, indicating minimal correlation and maximizing diversity gain. For opposite elements, ECC is 0.003, highlighting even lower correlation and enhancing antenna system diversity. These low ECC values ensure nearly independent behavior of antenna elements, crucial for diversity techniques in MIMO systems. Reference^6^ presents a different four-element MIMO system design featuring L-monopole antennas. This alternative design highlights the versatility of MIMO antenna configurations, meeting diverse application scenarios and performance needs in wireless systems. Reference^6^ presents an antenna with an impressive 58.6% impedance bandwidth (IBW) from 2.24 to 4.94 GHz, maintaining a compact 40 × 40 mm^2^ size. With a minimum isolation of 11 dB and ECC values below 0.1, it ensures effective isolation and low correlation between elements. This design demonstrates MIMO antennas’ ability to operate across wide frequency ranges while minimizing size constraints. Reference^7^ introduces a four-element MIMO antenna with half-circle shape monopoles measuring 110 × 60 mm^2^. It achieves a bandwidth of 230 MHz and a minimum isolation of 11 dB between elements. This design showcases the diversity of antenna configurations and shapes for specific performance goals, accommodating various size and frequency requirements. Reference^8^ introduces a new four-element MIMO antenna with symmetrical dipoles and integrated baluns, likely advancing performance, size, or other characteristics compared to prior designs. Detailed specifications like bandwidth, isolation, and size are not available here. However, the work effectively isolates parallel elements with the same polarization by carefully choosing their distance. The chosen distance minimizes interference and crosstalk between adjacent elements, ensuring isolation. Additionally, polarization diversity achieves isolation between adjacent elements with orthogonal polarization. Combining physical separation and polarization diversity, this dual approach enhances signal integrity and mitigates interference, enabling reliable wireless communication across diverse environments. Numerous MIMO antenna designs, like the one in Ref.^9^ introducing an 8-element MIMO antenna, incorporate a greater number of ports. This enables support for higher-order MIMO configurations, enhancing spatial multiplexing and diversity gains. More antenna elements exploit additional spatial dimensions, improving spectral efficiency and reliability. While such designs may involve complex structures and require sophisticated signal processing, they promise greater performance improvements for applications needing higher data rates and increased network capacity. MIMO antennas commonly use linear polarization (LP) or circular polarization (CP), with CP often favored for its advantages.

Circular polarization (CP) effectively mitigates multipath fading, where signals arrive via multiple paths, preventing signal cancellation or distortion. Its ability to maintain signal integrity in such conditions suits challenging propagation environments. CP also performs well in adverse weather, being less susceptible to degradation from rain, snow, or fog compared to linear polarization. This ensures more reliable communication links even in inclement weather. Furthermore, CP enables acceptable mobility in wireless systems. Circular polarization reduces the criticality of receiving antenna orientation relative to the transmitting antenna, offering flexibility in device orientation and movement without significant signal degradation. These advantages contribute to CP’s ability to deliver high-quality communication service across various wireless scenarios, making it a preferred choice for many MIMO antenna applications^10,11^. Contemporary research emphasizes designing circularly polarized (CP) antennas with diverse techniques to achieve satisfactory radiation properties. Among these, employing the Tai Chi shape in antenna configuration has gained attention^12,13^. Inspired by the ancient Chinese symbol representing yin and yang forces, the Tai Chi shape offers unique geometric properties to enhance antenna performance. Researchers incorporate this shape to improve circular polarization characteristics, including axial ratio and impedance bandwidth. Studies incorporating the Tai Chi shape in CP antenna designs investigate its influence on radiation patterns, gain, and efficiency. Researchers optimize antenna structure and geometry to leverage the shape’s symmetry and balance for desired performance. These innovative techniques, including Tai Chi shape utilization, reflect ongoing efforts to advance CP antenna design and enhance MIMO system capabilities across wireless applications^12–16^ adopts an innovative approach with an asymmetric microstrip feed line and a parasitic patch to realize a complete Tai Chi-shaped antenna structure. It achieves impressive impedance bandwidths (IBWs) from 3.1 to 4.58 GHz and 4.97–6.53 GHz, along with axial ratio bandwidths (ARBWs) from 3.26 to 4.42 GHz and 5.45–6.63 GHz. These wide bandwidths indicate effective operation across multiple frequency bands while maintaining circular polarization. Reference^13^ presents a CP array antenna with semi-fractal radiation patches featuring Tai Chi-shaped elements, covering 5.3–6.8 GHz, with an ARBW from 5.4 to 6.6 GHz. This integration enhances performance and maintains circular polarization within the desired frequency range. These studies demonstrate the effectiveness of integrating the Tai Chi shape in CP antenna design, showcasing its potential for achieving wide bandwidths and desirable polarization characteristics crucial for modern wireless systems. Moreover, the Tai Chi shape extends beyond CP antenna design applications. In reference^17^, researchers utilize it for radar cross-section (RCS) reduction, manipulating electromagnetic wave scattering to minimize antenna radar signatures. This application highlights the versatility of the Tai Chi shape in antenna engineering, beyond traditional metrics like impedance bandwidth and axial ratio. Similarly, in Ref.^18^, the Tai Chi-shaped element enables a dual-band antenna covering frequencies from 2.4 to 2.49 GHz and 5.07–5.88 GHz, catering to multiple wireless communication standards or applications. Its integration facilitates dual-band operation while maintaining compactness and desirable radiation characteristics. These examples illustrate the Tai Chi shape’s diverse applications and benefits in antenna design, offering a versatile and effective tool for engineers to explore across various functionalities, from circular polarization to RCS reduction and dual-band operation. A multiband hybrid Multiple Input Multiple Output (MIMO) dielectric resonator antenna (DRA) designed for Sub-6 GHz 5G and WiFi-6 applications. The focus is on enhancing communication performance by covering multiple frequency bands and providing efficient signal transmission in the evolving 5G and WiFi environments^19^. A MIMO antenna system with four ports designed for Sub-6 GHz 5G New Radio (NR) communication. The study emphasizes the importance of achieving high isolation between ports to enhance overall performance, ensuring minimal interference and improved signal quality in 5G communication systems. Both studies contribute to advancing antenna technology for next-generation wireless communication^20^. Circular polarization can be generated through various techniques depending on the specific application and frequency range. One of the most common methods is the use of a quarter-wave plate (QWP), which creates a 90° phase shift between orthogonal light components, converting linear polarization into circular polarization. This approach is widely employed in optical systems^21^. Circular polarizing filters, commonly used in photography, combine a linear polarizer with a QWP to selectively transmit circularly polarized light^22^. Spiral phase plates, which shape laser beams by introducing a helical wavefront, are another technique^23^. Reflective surfaces, such as metallic or dielectric reflectors, can also induce circular polarization by causing differential phase shifts^4^. At radio and microwave frequencies, specially designed antennas, like helical and patch antennas, are used in satellite communications and radar systems^24,25^. Recently, metamaterials with subwavelength structures have emerged as a versatile tool for manipulating polarization^26–30^.

The literature shows that MIMO technology significantly enhances wireless communication through multiple antennas and array configurations, improving data speed, reliability, and spectral efficiency. However, maintaining sufficient isolation between antennas remains a critical challenge in compact MIMO systems. As the number of antennas increases in a confined space, the risk of interference and crosstalk also rises potentially degrading system performance. Ensuring adequate isolation between antennas is essential to prevent these negative effects and ensure reliable communication. Although numerous studies have explored various MIMO antenna designs and isolation techniques, such as orthogonal placement, parasitic structures, and polarization diversity, achieving high isolation in compact designs while maintaining performance and compactness remains unresolved. Overcoming this challenge is vital to fully realizing MIMO’s potential and delivering robust wireless solutions, especially in space-constrained applications.

This article addresses the identified research gap by introducing a flexible four-element MIMO antenna designed explicitly for X-band applications, offering a significant advancement in achieving high isolation in compact MIMO systems. The proposed antenna operates over the 10.6–11.9 GHz spectrum, featuring a modified E-shaped radiating patch and a unique feeding structure that enhances performance. Printed on a flexible felt substrate with a compact size of 60 mm × 60 mm × 1 mm, the antenna maintains circular polarization across the desired bands with an impressive axial ratio bandwidth of 1.2 GHz. The antenna achieves a maximum gain of up to 8 dBi and demonstrates excellent MIMO performance, validated through parameters like ECC, DG, CCL, and MEG. Additionally, the antenna’s robustness under various bending scenarios confirms its suitability for conformal X-band MIMO applications. Through innovative design and comprehensive testing, this work successfully addresses the challenges of isolation in compact MIMO systems, contributing to the advancement of reliable, high-performance wireless communication solutions.

The major contributions of the article are listed below.

- Introduction of a Flexible Four-Element MIMO Antenna for X-band applications operating over the 10.6–11.9 GHz spectrum.
- Incorporation of a modified E-shaped radiating patch and unique feeding structure to enhance performance and achieve high isolation.
- The antenna is printed on a flexible substrate of 60 mm × 60 mm × 1 mm, suitable for conformal applications.
- The antenna maintains circular polarization with a 1.2 GHz axial ratio bandwidth.
- The antenna achieves a maximum gain of up to 8 dBi.
- The antenna’s performance is stable under various bending conditions, proving its suitability for flexible applications.

The article is structured as follows: “Antenna design” discusses the proposed four-element MIMO antenna and its analysis. The results and discussion are presented in “Measured results and discussions”, followed by the conclusion in “Conclusion”.

## Antenna design

**Fig. 1:**
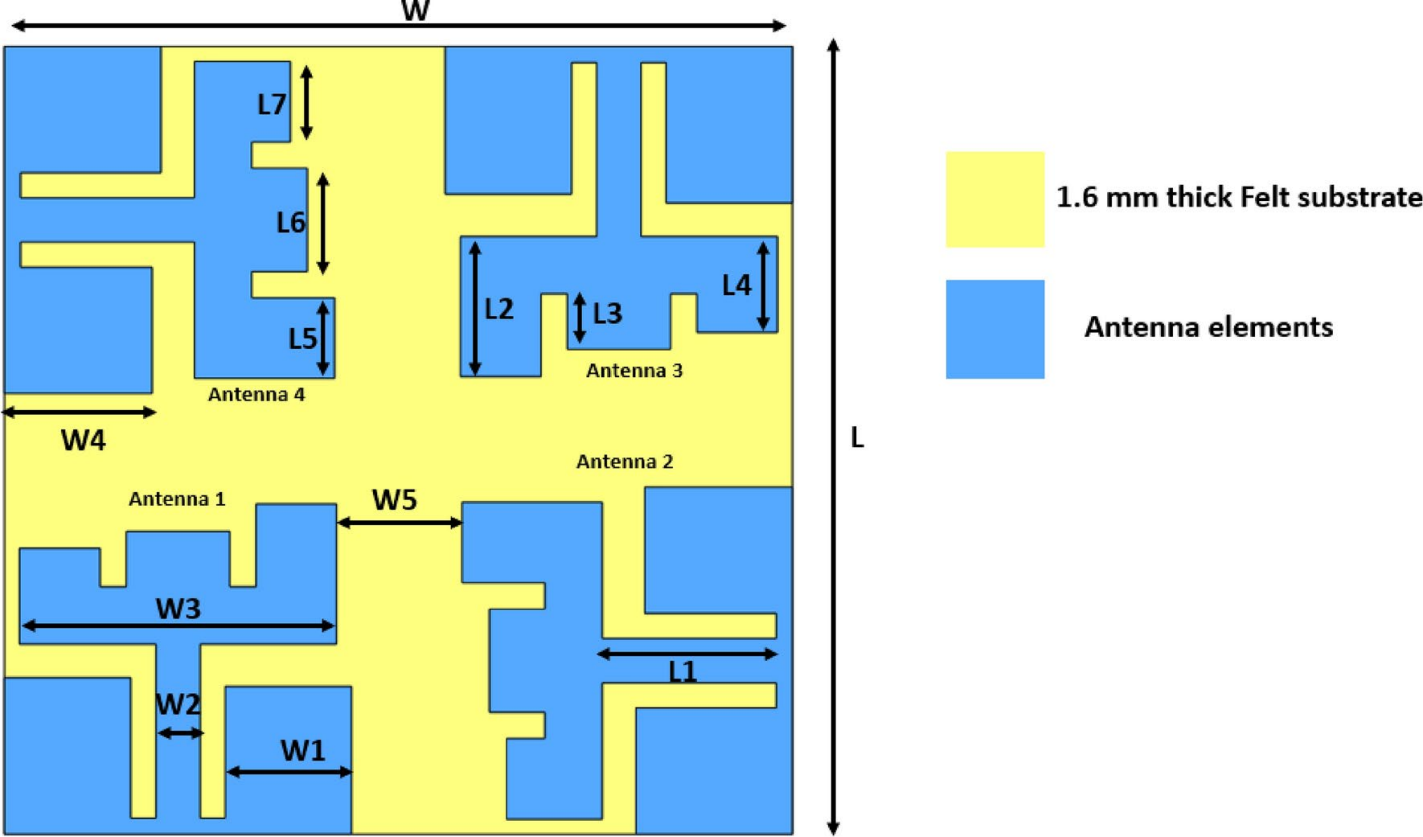
Structure and dimension of the proposed conformal MIMO antenna.

The proposed four-port antenna, illustrated in Fig. 1, features antenna elements positioned at the four corners of a flexible felt substrate with a dielectric constant of 1.2 and a tangential loss of 0.02, arranged to radiate electromagnetic waves orthogonally. The overall dimensions of the MIMO antenna measure 60 mm × 60 mm × 1 mm, with each antenna comprising a modified E-shaped radiating element with unequal arms fed by a 50-Ω feeding strip. Two rectangular patches are strategically placed on two sides of the antenna to improve impedance matching and broaden the bandwidth. The substrate’s backside is covered by a full ground plane. Detailed dimensions of the proposed four-port flexible MIMO antenna are provided in Table 1. The E-shaped symbol in the antenna design is motivated by its versatility in achieving compact, wide-band, and customizable performance. This shape supports the efficient use of space on the substrate, enabling broad resonances and covering various frequency bands, which is particularly beneficial for communication systems requiring wideband or multiband capabilities. Additionally, the E-structure enhances bandwidth and gain through its adjustable geometry, allowing fine-tuning of frequencies by modifying the arm lengths and widths. Its symmetry aids in achieving balanced radiation patterns and improved gain while remaining straightforward to fabricate. This makes it suitable for both traditional and flexible materials, supporting miniaturization trends and innovative applications, such as wearable antennas and space-constrained devices.

**Table 1:** Various dimensions of the proposed antenna.

Parameters	L	L1	L2	L3	L4	L5	L6	L7	W	W1	W2	W3	W4	W5
Values (mm)	60	14.5	10.65	4.2	7.28	6.13	7.875	6.13	60	9.62	3.375	24.1	11.25	9.57

**Fig. 2:**
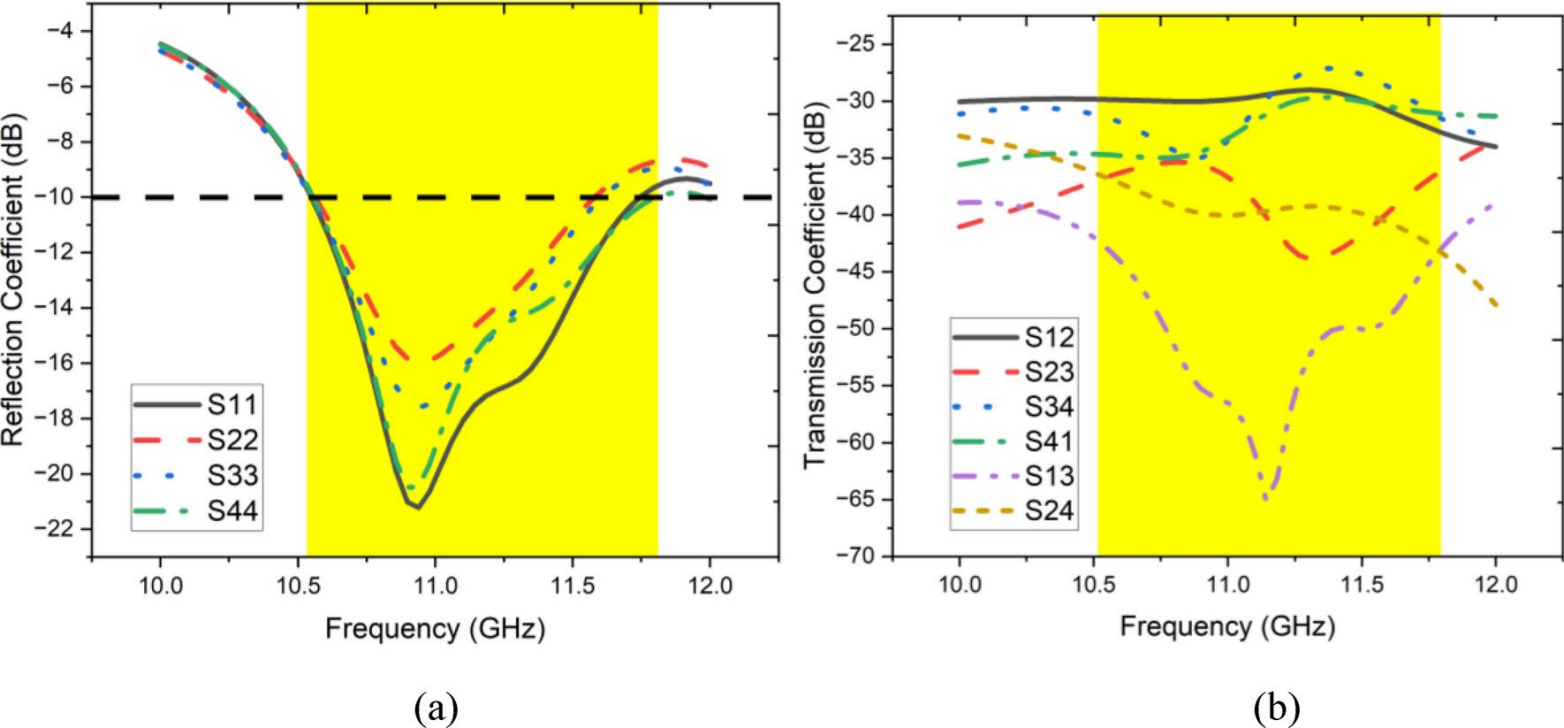
Simulated (**a**) Reflection coefficient, (**b**) Transmission coefficient.

Figure 2a,b display the proposed four-element antenna’s simulated reflection and transmission coefficients, respectively. Notably, each antenna element within the MIMO system covers a spectrum from 10.6 to 11.8 GHz with a bandwidth of 1.2 GHz, effectively occupying a significant portion of the X-band, which ranges from 9 to 12 GHz. The transmission coefficient in Fig. 2b shows that the isolation between antenna elements exceeds 29 dB across the operational spectrum, attributed to the orthogonal arrangement of antenna elements. This substantial enhancement in isolation significantly augments the performance of the MIMO antenna in terms of gain, ECC, and CCL.

**Fig. 3:**
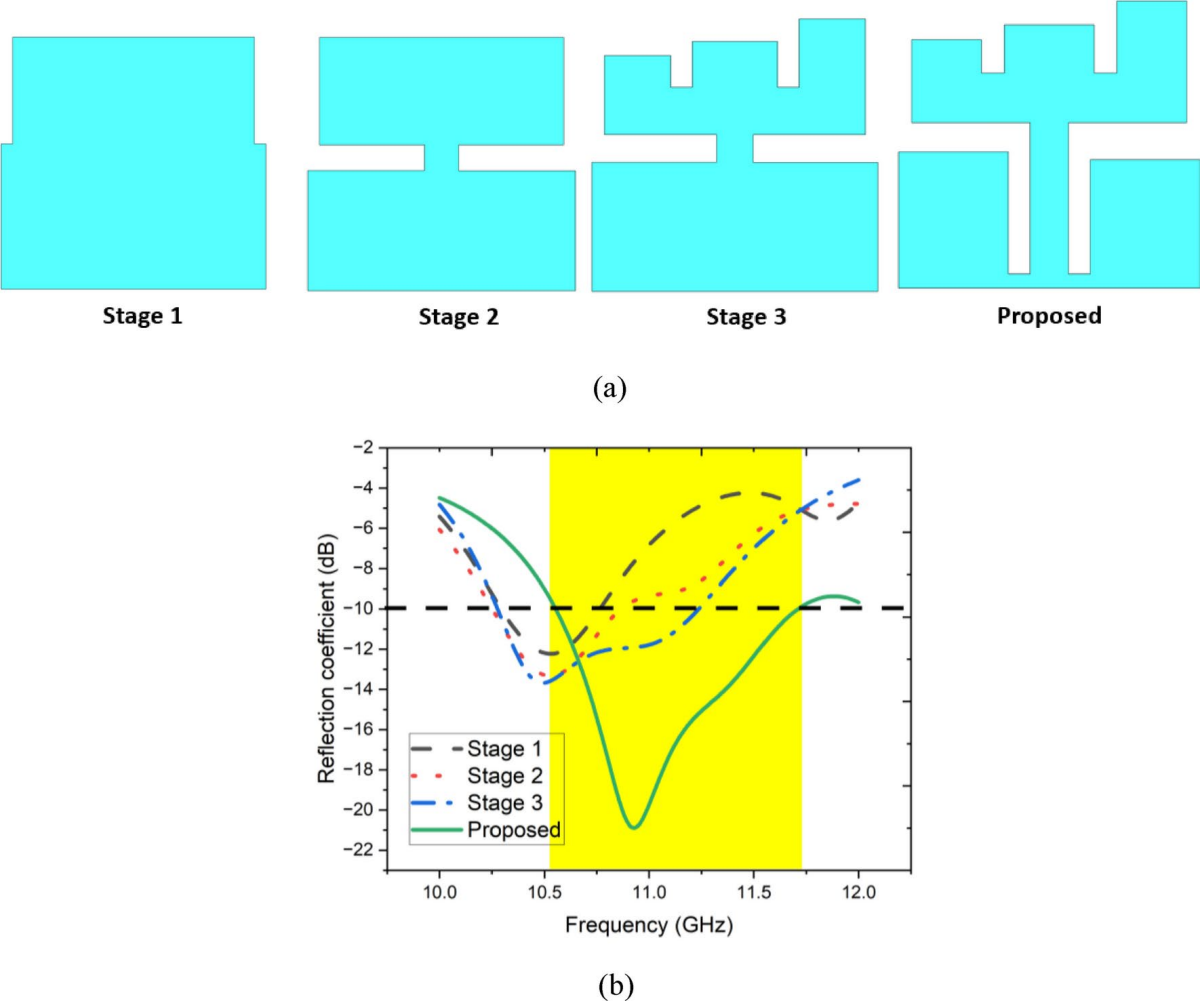
(**a**) Design iterations of the antenna 1 and its (**b**) reflection coefficient.

Figure 3a illustrates the design evolution of the proposed antenna. The antenna is initially composed of two rectangular sections arranged one above the other, as illustrated in Stage 1. Figure 3b illustrates the reflection coefficients for each design iteration. The antenna resonates at 10.5 GHz with a limited bandwidth in Stage 1. The etching of two rectangular segments initiated stage 2 to improve performance. As illustrated in Fig. 3b, this modification led to a modest increase in operating bandwidth. Stage 3 was established by converting the upper rectangular patch into an E-shaped patch with unequal limbs to enhance performance. Compared to Stage 2, the bandwidth of the Stage 3 antenna was marginally increased. In the final stage, the antenna evolved from Stage 3 by vertically eliminating two rectangular segments from the bottom radiating patch, as illustrated in Fig. 3a. This modification produced two rectangular openings at the base of the patch, which enabled wide-band operation within the 10.6–11.7 GHz frequency range with a reflection coefficient of − 22 dB at 10.9 GHz.

**Fig. 4:**
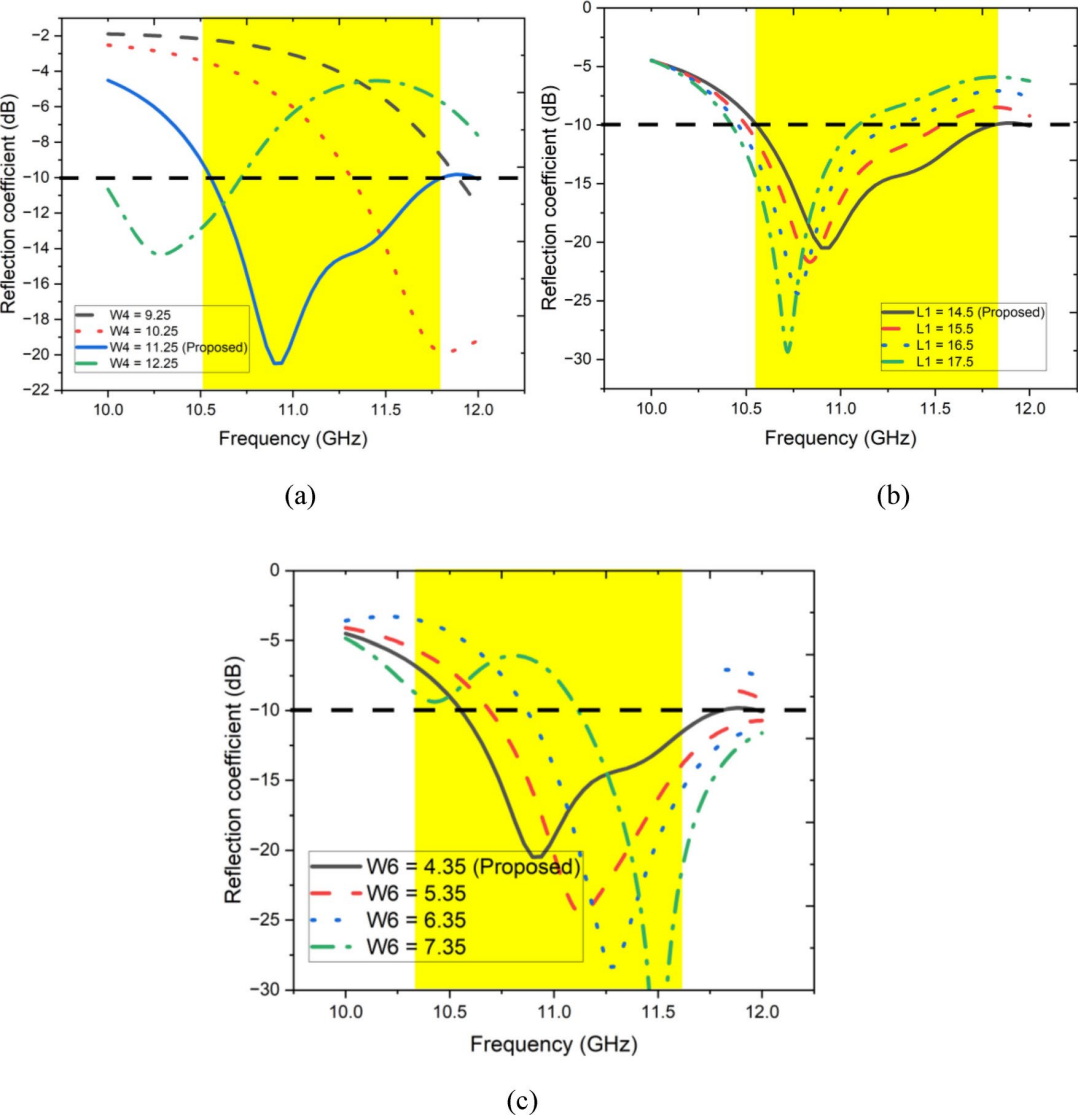
Parametric study when analyzing (**a**) W4, (**b**) L1, (**c**) W6.

**Fig. 5:**
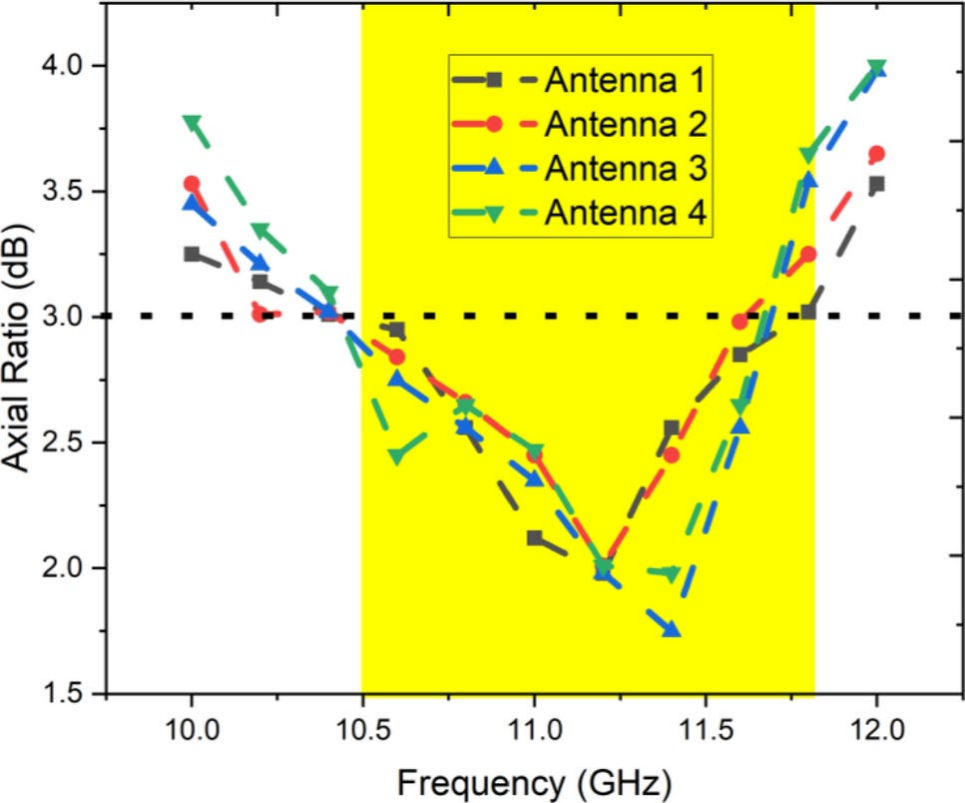
Axial ratio of proposed MIMO antenna.

This section provides a parametric report on the proposed MIMO antenna, as shown in Fig. 4a–c. In Fig. 4a, the length of the feeding strip connected to the rectangular strip (W4) varies from 9.25 to 12.25 mm in 1 mm increments. Decreasing the value of W4 shifts the center frequency to a higher frequency band. Thus, the length of W4 affects the operating spectrum of the proposed antenna. Similarly, the length of the feeding strip (L1) varies from 14.5 to 17.5 mm, with the corresponding reflection coefficient shown in Fig. 4b. Changes in L1 affect the impedance matching of the antenna in the desired operating band, with optimal performance achieved at 14.5 mm. When the height of the E-shaped patch is changed from 4.35 to 7.35 mm, the frequency shifts to the right side of the spectrum, as depicted in Fig. 4c. The required band of operation at 4.35 mm is achieved with a reflection coefficient of − 21 dB at a center frequency of 11 GHz (Fig. 5).

**Fig. 6:**
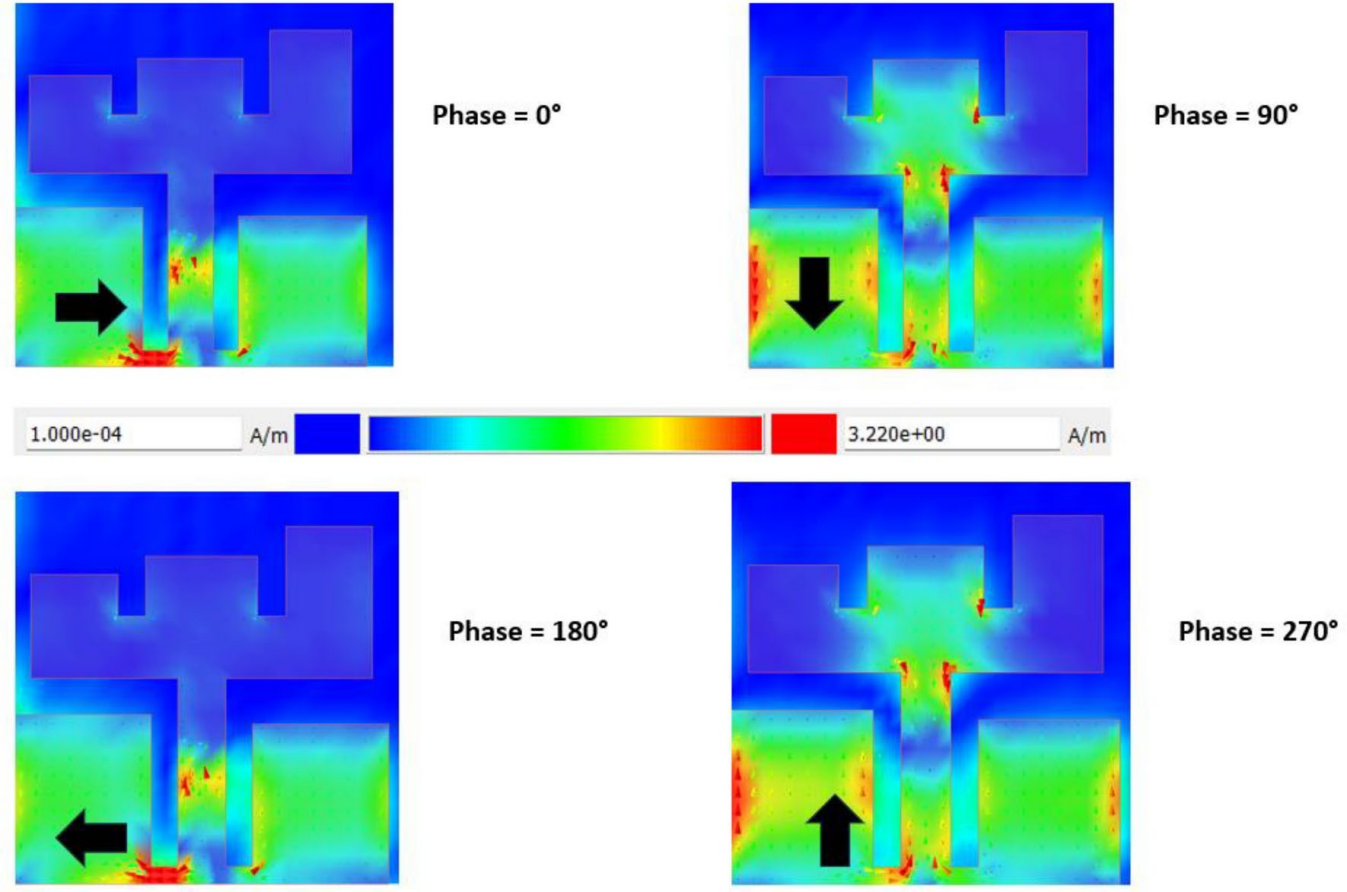
Surface current distribution of antennas 1 at various phase angles.

**Fig. 7:**
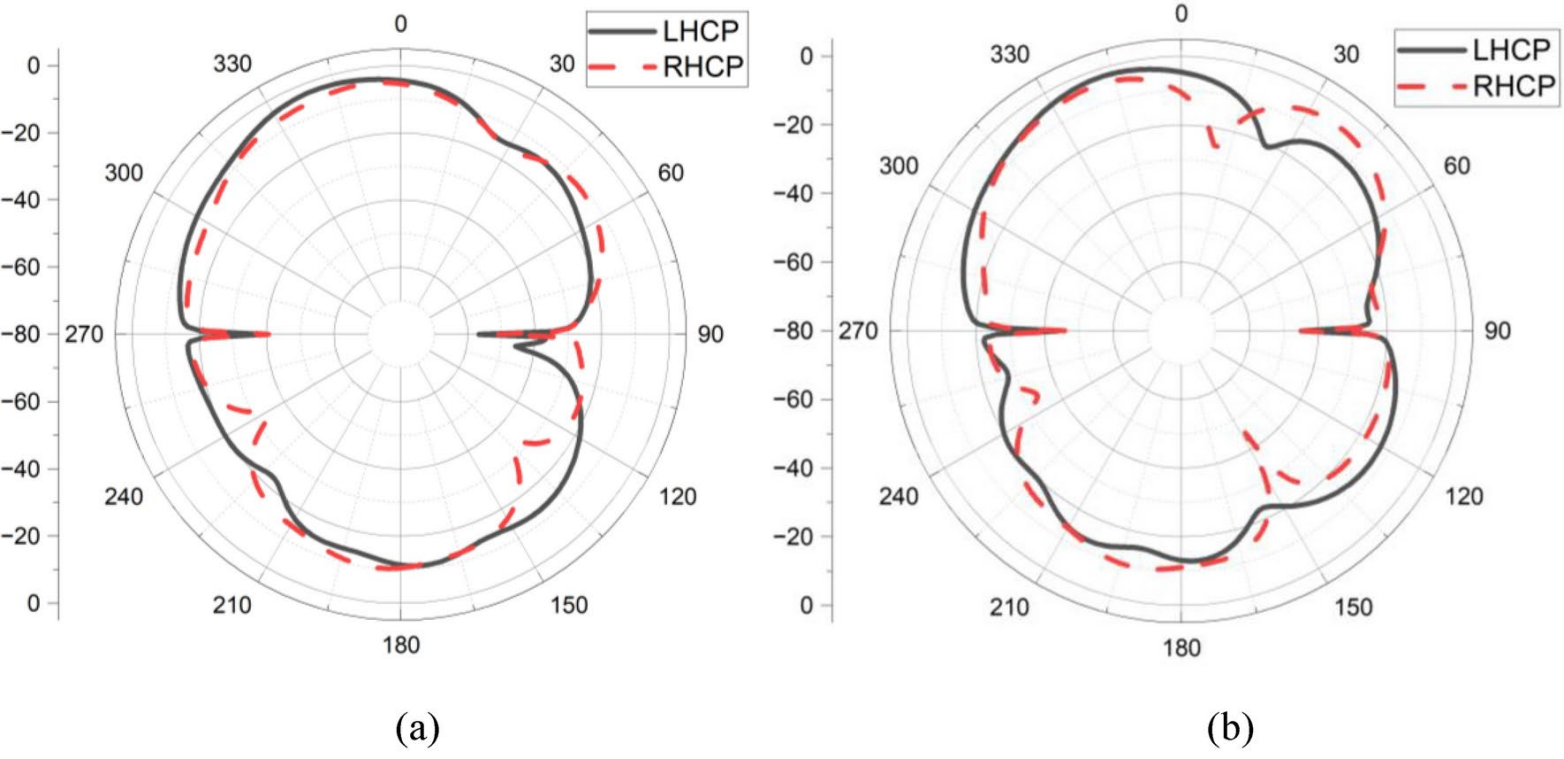
LHCP and RHCP of (**a**) Antenna 1, (**b**) Antenna 2.

Furthermore, Fig. 6 depicts the surface current distribution on the radiating patch at the frequency corresponding to the lowest point on the AR curve (11 GHz), as depicted in Fig. 5 when port-1 is excited. The surface currents, with phases of 0°, 90°, 180°, and 270°, clearly exhibit a clockwise rotation on the patch. The normalized radiation patterns for the right-hand circular polarization (RHCP) and left-hand circular polarization (LHCP) of the MIMO antenna are illustrated in Fig. 7. These patterns are shown explicitly at frequencies of 9.8, 10, and 10.2 GHz, focusing on the zy-plane with Ⴔ = 0°. It’s important to note that all electromagnetic simulations conducted in this study utilized Keysight’s Advanced Design System (ADS) EM simulator.

**Fig. 8:**
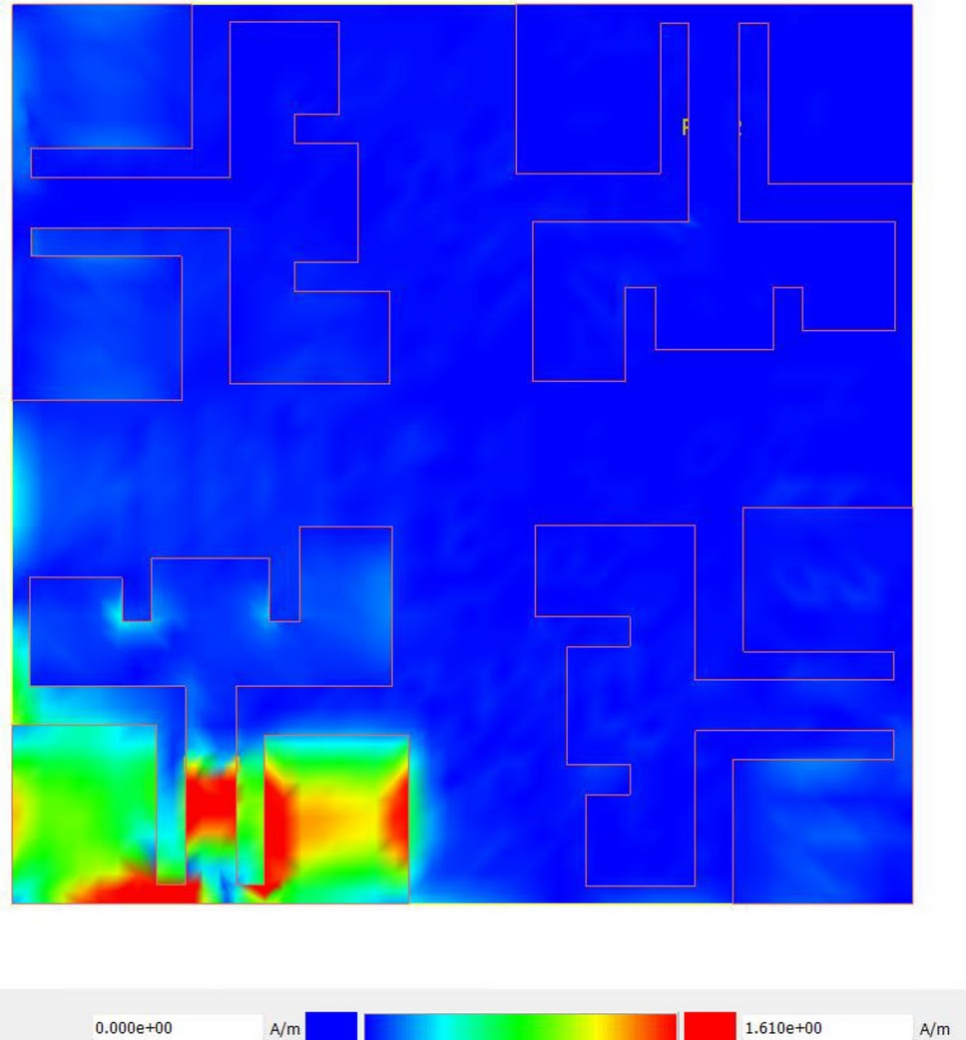
Surface current distribution of MIMO antenna when antenna 1 is excited.

The surface current distribution of the proposed antenna at 11 GHz is illustrated in Fig. 8. The maximum current distribution is observed along the middle portion of the feeding strip and the right-side rectangular stub, indicating that the bottom portion of the radiating element affects both the operating frequency and impedance matching. Isolation between the MIMO antenna elements is enhanced by minimizing the current coupling to the adjacent antenna elements. In this figure, the majority of the surface current is confined to the lower left area, while the rest of the antenna structure, especially near other antenna elements, shows significantly lower current intensity. This demonstrates effective isolation between the ports, as the excitation at Port 1 does not result in substantial current flow on the other elements.

## Measured results and discussions

**Fig. 9:**
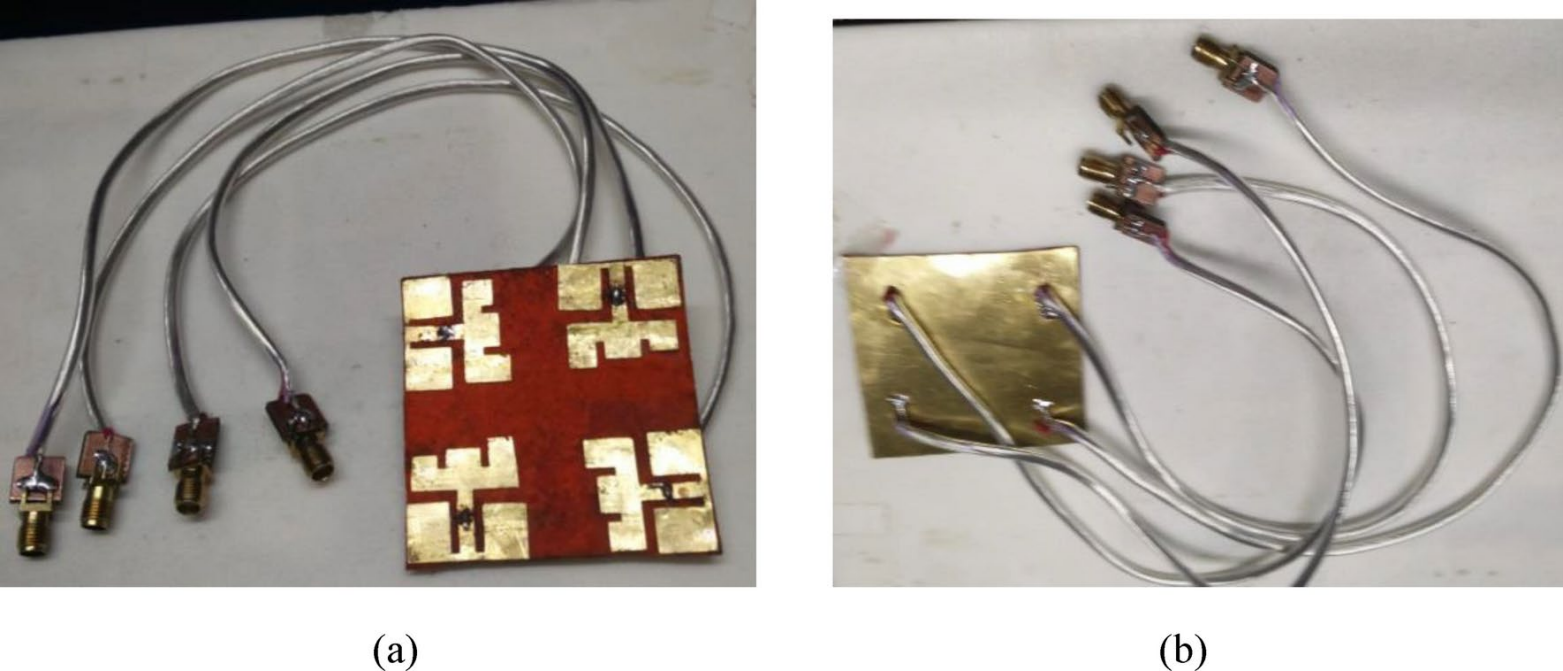
Fabricated prototype (**a**) front view, (**b**) rear view.

**Fig. 10:**
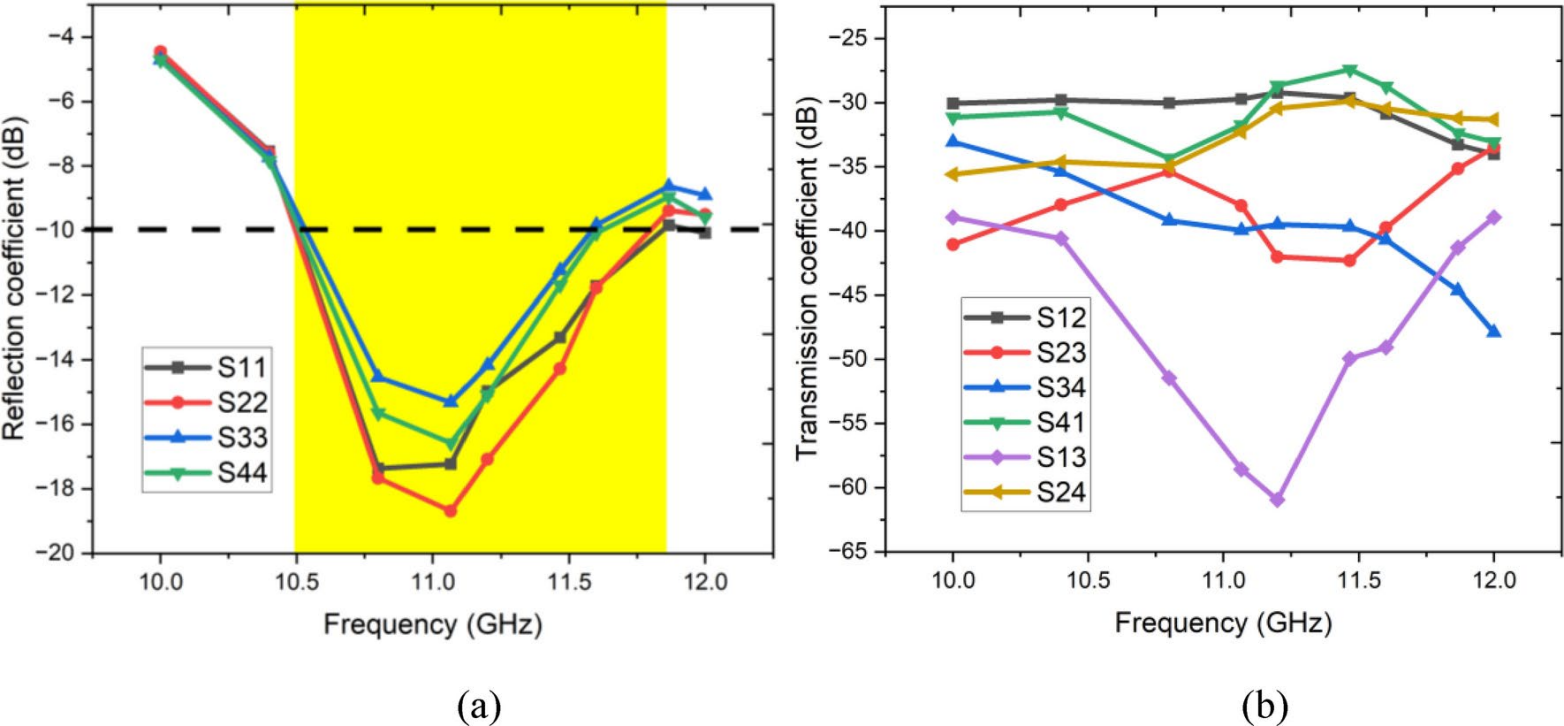
Measured (**a**) reflection coefficient, (**b**) transmission coefficient.

Figure 9a,b respectively depict the front and rear views of the fabricated four-port antenna. Each antenna element is connected to a 50-Ω SMA connector, and during measurement, all the elements have been terminated by a 50-Ω terminator except the antenna being tested. S-parameters of the antenna are measured using Keysight’s Vector Network Analyzer (VNA), and radiation patterns are measured in an anechoic chamber. Figure 10a,b depict the measured reflection and transmission coefficients. Figure 10a shows that all the elements are resonating in the desired band of the spectrum from 10.6 to 11.9 GHz on a 10 dB impedance bandwidth. Similarly, the transmission coefficients are better than 25 dB over the operating spectrum, as depicted in Fig. 10b. Moreover, it is noted that both measured reflection and transmission coefficients closely coincide with the simulated results.

**Fig. 11:**
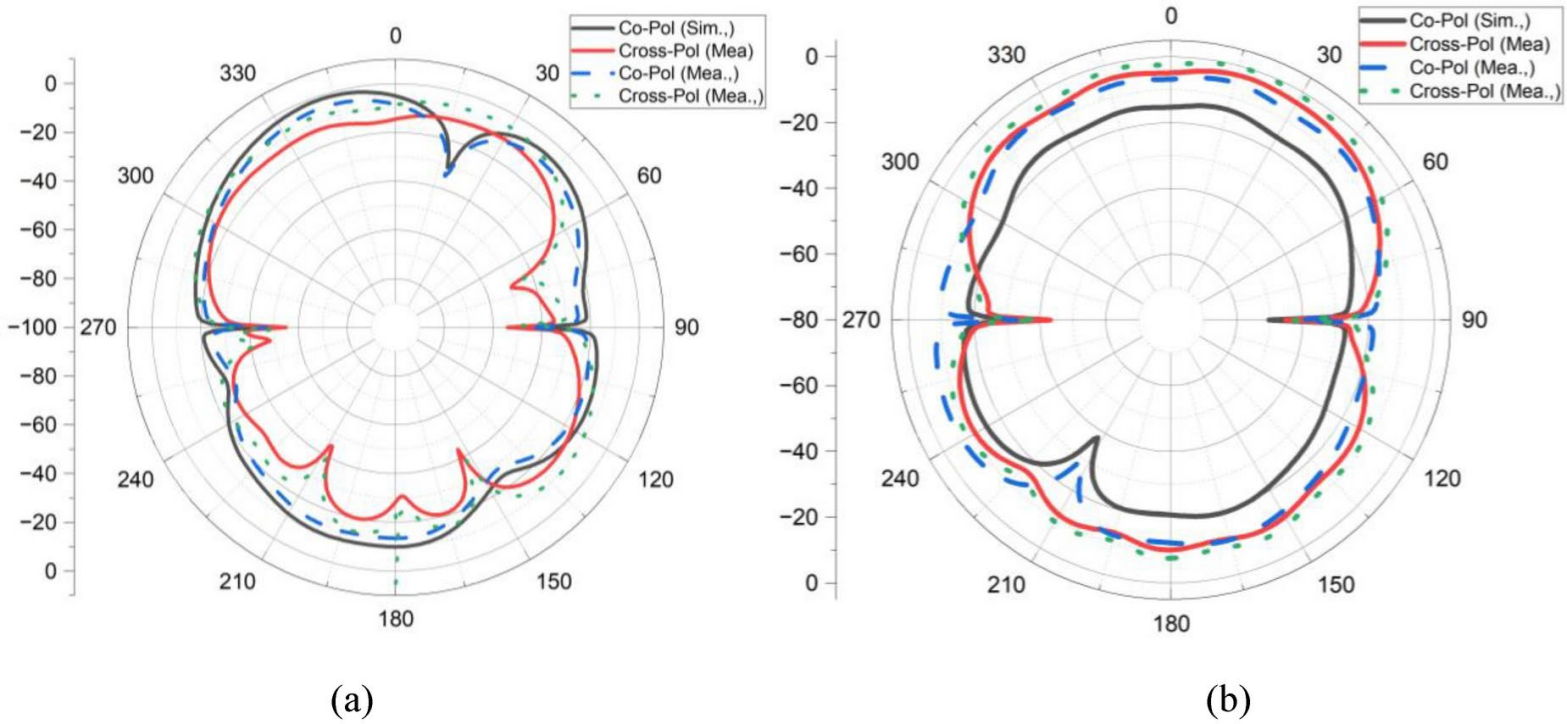
Simulated and measured far-field pattern (**a**) Antenna 1, (**b**) Antenna 2.

The measured and simulated far-field patterns of antenna 1 and 2 are depicted in Fig. 11a,b, respectively. It is observed that both simulated and measured co-polarization and cross-polarization patterns agree well with each other. Also, it is noted that both antenna elements exhibit independent electromagnetic propagation due to the orthogonal arrangements of the antenna elements.

**Fig. 12:**
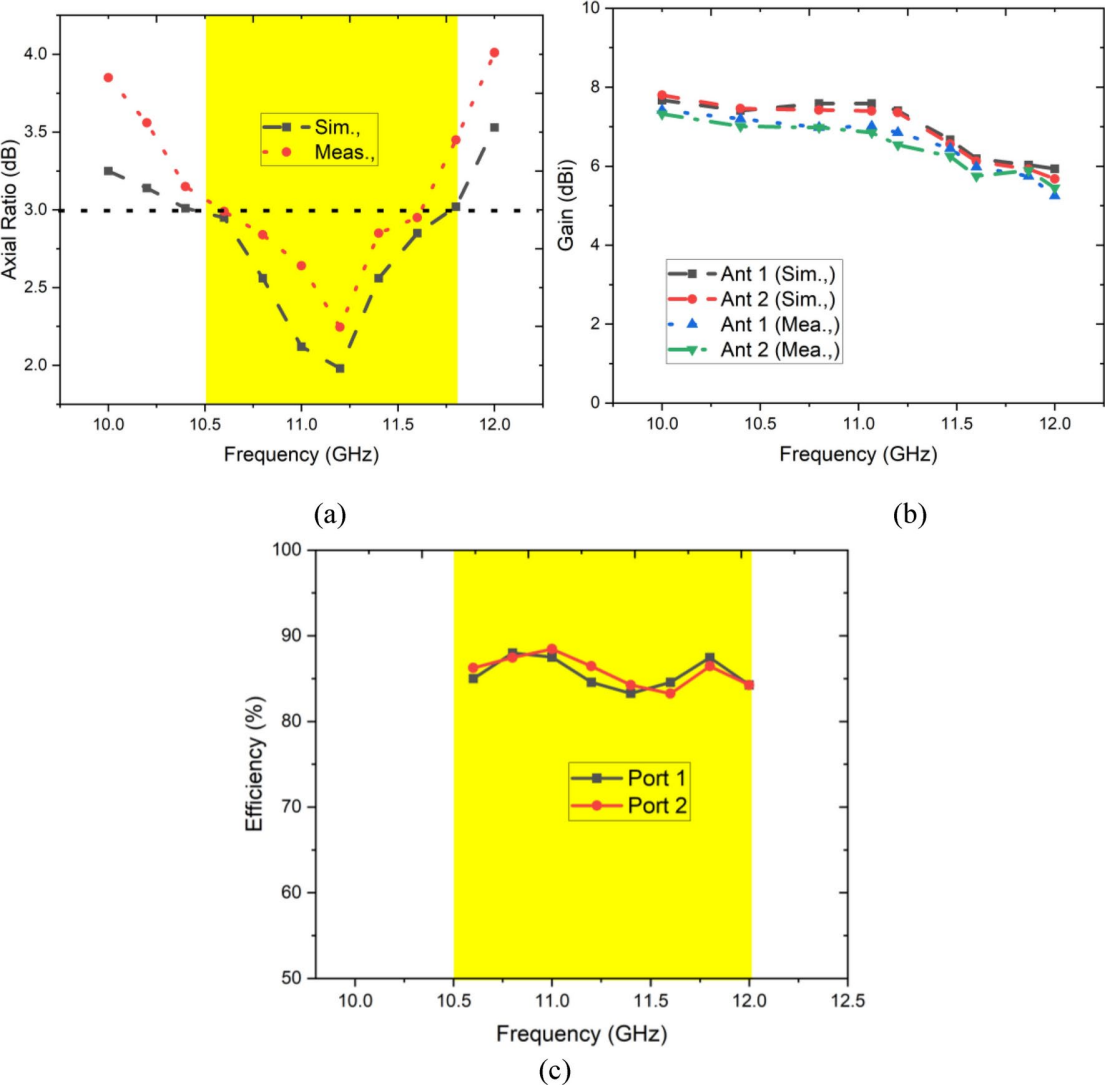
Simulated and measured (**a**) axial ratio, (**b**) gain, (**c**) Efficiency.

The simulated and measured axial ratio of the antenna 1 is depicted in Fig. 12a. Antenna 1 exhibits an overall ARBW (< 3 dB) of 1.18 GHz, sufficient for excellent circular polarization for X-band applications. Antenna 1 and 2 achieved peak gain up to 7.98 dB at 11 GHz, and it varies between 6.2 and 8 dBi over the operating spectrum as shown in Fig. 12b. The achieved gain is comparatively better than the other antennas reported in the literature.

Figure 12c presents the efficiency plot of a proposed antenna design, showing the performance of two ports (Port 1 and Port 2) across a frequency range from 10.0 to 12.5 GHz. For both ports, the efficiency values remain relatively stable within this operating range, consistently staying above 85%, with slight fluctuations around 90%. This high and stable efficiency across the specified frequency band indicates minimal losses and good radiation performance in the selected band.

### Conformability analysis

**Fig. 13:**
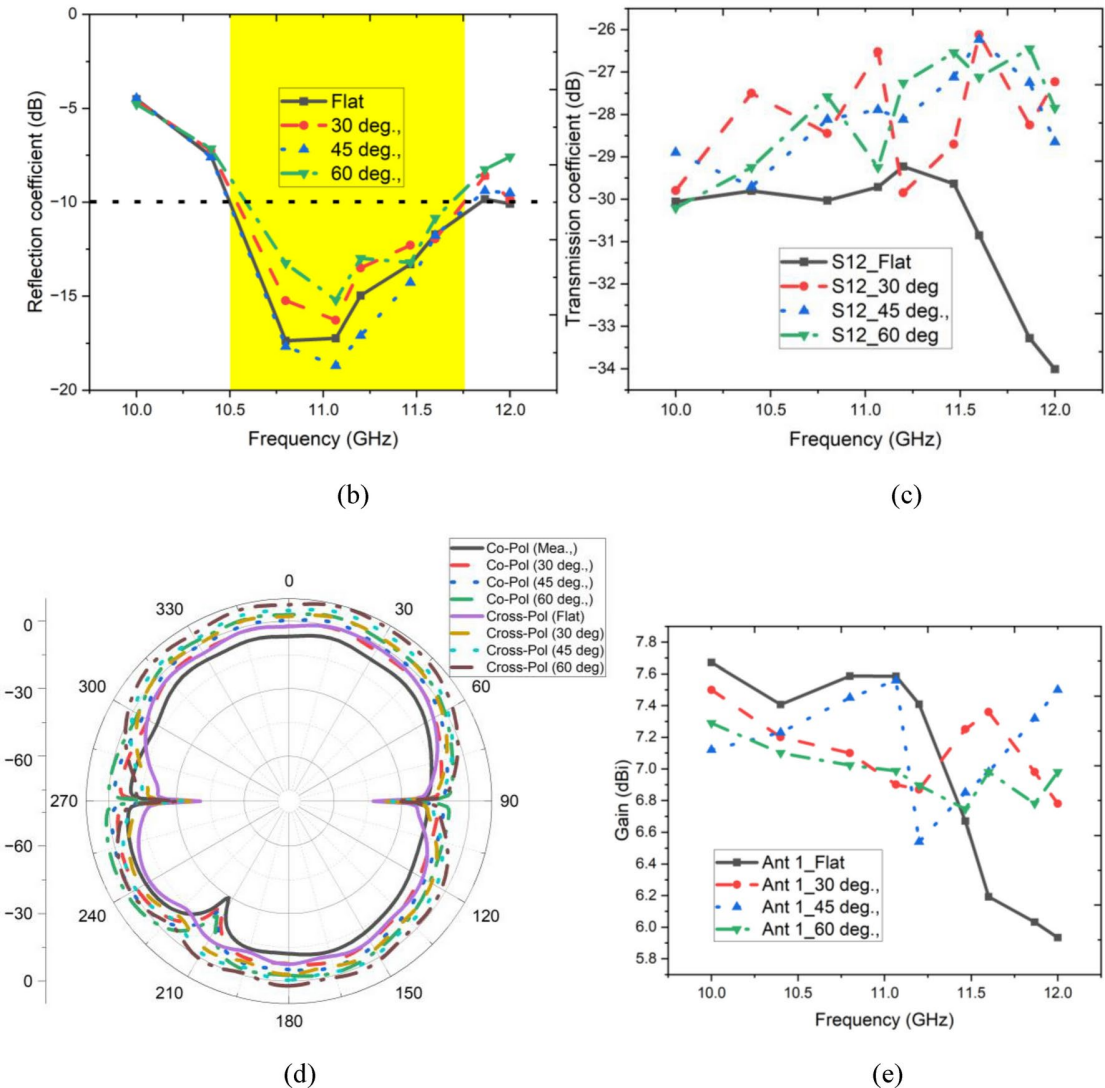
Conformal analysis of antenna 1 (**b**) Reflection coefficient, (**c**) transmission coefficient, (**d**) far-field pattern, (**e**) gain.

The durability and reliability of the antenna were evaluated through a sequence of bending tests. These tests included precise bending at angles of 30°, 45°, and 60° as illustrated in Fig. 13a. Such rigorous examination is essential for conformal antennas, ensuring their optimal performance on irregular surfaces.

Consistently, the antenna exhibited a reflection coefficient within optimal parameters across the spectrum (10.6–11.9 GHz), indicating exceptional impedance matching, as shown in Fig. 13b. The design ensured significant isolation between its ports, better than − 25 dB, as shown in Fig. 13c, effectively minimizing interference even under bending conditions. As depicted in Fig. 13d, the two-dimensional radiation pattern during flat conditions and other bending scenarios has similar patterns along yz planes, ensuring that the conformability of the proposed antenna does not affect any of the performance metrics of the antenna system. Stable gain has been maintained across the desired frequency bands, as illustrated in Fig. 13e, a pivotal aspect ensuring the antenna’s dependable signal propagation and reception performance.

### MIMO performance

A complete set of experiments has been carried out to thoroughly assess the performance of the proposed system’s Multiple Input Multiple Output (MIMO) capabilities, focusing on several MIMO performance metrics. These factors are crucial in evaluating the effectiveness of antennas in realistic, real-world situations^22–24^. The metrics used in this analysis include Channel Capacity Loss (CCL), Mean Effective Gain (MEG), Diversity Gain (DG), and Envelope Correlation Coefficient (ECC). Figure 10 illustrates the ECC, TARC, CCL, and DG characteristics of the proposed MIMO antenna,

1 $$ECC = \frac{{\left| {\left( {\int {\int_{0}^{4\pi } {A_{i} \left( {\theta ,\varphi } \right)XA_{j} \left( {\theta ,\varphi } \right)} } } \right)d\Omega } \right|^{2} }}{{\left| {\int {\int_{0}^{4\pi } {A_{i} \left( {\theta ,\varphi } \right)} } } \right|^{2} d\Omega \left| {\int {\int_{0}^{4\pi } {A_{j} \left( {\theta ,\varphi } \right)} } } \right|^{2} d\Omega }}$$

**Fig. 14:**
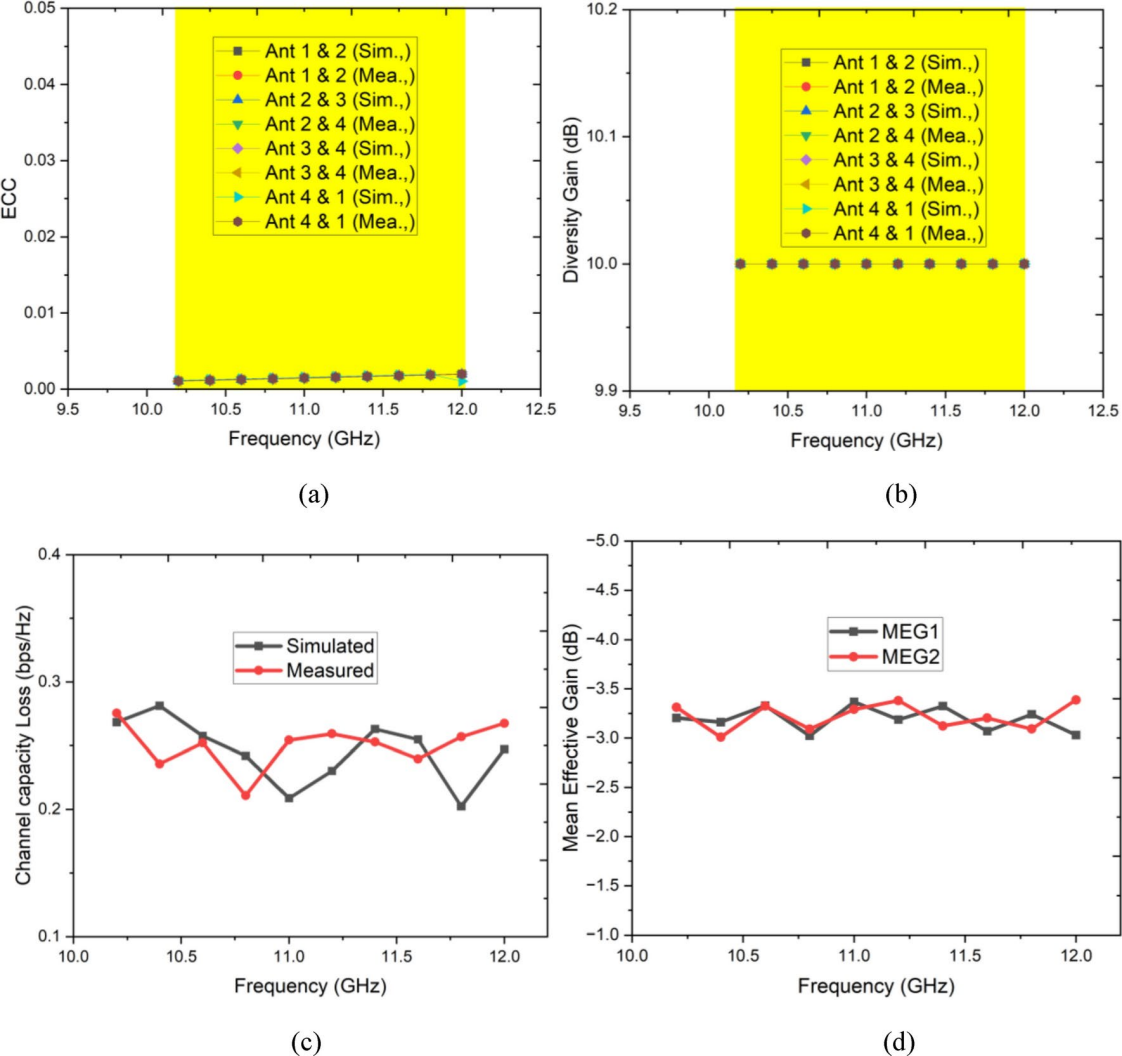
MIMO performance (**a**) ECC, (**b**) Diversity gain, (**c**) CCL, (**d**) MEG.

The ECC value is found to be close to zero, as shown in Fig. 14a. In contrast, the DG value, computed using Eq. (2) for the corresponding bands, is around 10 dB (see Fig. 14b).

2 $$Diversity\;Gain \left(DG\right)=10\sqrt{1-{ECC}^{2}}$$

The CCL (Coupling Loss Coefficient) is calculated using Eqs. (3) and (4) and is found to be less than 0.4, as indicated in Fig. 14c.

3 $$Channel\;Capacity\;Loss \left(CCL\right)= -{\text{log}}_{2}\text{det}({\gamma }^{r})$$

4 $${\gamma }^{r}=\left(\begin{array}{ccc}{ECC}_{11}& \cdots & {ECC}_{14}\\ \vdots & \ddots & \vdots \\ {ECC}_{41}& \cdots & {ECC}_{44}\end{array}\right)$$

The mean effective gain of antenna 1 and 2 is depicted in Fig. 14d. Both the antenna elements have an MEG of − 3 dB which is the optimal margin for better performance of the MIMO antenna system.

The proposed antenna represents an innovative advancement in antenna design as listed in Table 2, distinctly surpassing its counterparts. Operating within the 10.3–11.9 GHz frequency range, it offers exceptional performance metrics, including a bandwidth of 14.4% and outstanding isolation exceeding < − 29 dB. With a peak gain of 8.13 dBi, it efficiently boosts signals, all within a compact 60 × 60 mm form factor that seamlessly integrates into various systems. Utilizing flexible felt substrate instead of conventional materials enhances durability and signal integrity. Its minimal ECC rating of 0.001 underscores its precise engineering, minimizing errors for maximum data accuracy. Overall, this antenna not only sets new standards of excellence but also signifies a transformative era in antenna engineering, poised to shape the future of wireless communication with its unmatched performance and adaptability.

**Table 2:** Performance comparison of the proposed antenna with other antennas reported in the literature.

References	Operating frequency	Bandwidth (%)	Distance between antenna elements (mm)	Isolation (dB)	Peak gain (dBi)	The overall size of the antenna	Substrate used	ECC
^5^	2.32–2.95	23.9	22	< − 17	5.5	85 × 85	Rogers 4003c	0.008
^6^	2.70–4.94	58.6	NA	< − 11	4	40 × 40	FR4	0.1
^7^	2.2–2.43	9.9	13.76	< − 11	3.12	110 × 60	FR4	0.3
^8^	2.2–2.72	21.1	60,84.7	< − 15	8.37	3.14 × 802	FR4	0.01
^27^	7.58–8.04 and 9.23–10.79	5.9 & 15.6	47.5	< − 20	2.5	46.7 × 46.7	FR4	0.003
Proposed antenna	10.3–11.9	31.58 & 11.11	9.57	< − 29	8.13	60 × 60	Felt	0.001

## Conclusion

In conclusion, this article presented a versatile four-element antenna designed for X-band applications. The proposed antenna operated within the 10.6–11.9 GHz range, encompassing a significant portion of the X-band. It featured a single element composed of a modified E-shaped radiating patch, fed by a distinctive 50-Ω feeding structure with two rectangular stubs on either side. All elements were printed on a 60 mm × 60 mm × 1 mm flexible felt substrate, achieving circular polarization with an axial ratio bandwidth of 1.2 GHz, crucial for X-band applications. Practical testing confirmed the antenna’s optimal performance, with simulated and measured results in close agreement. Additionally, the antenna reached a maximum gain of 8 dBi within the desired band. Comprehensive MIMO parameter analysis, including ECC (< 0.01), DG (> 9.8 dB), CCL (< 0.3), and MEG (~ − 3 dB), validated its suitability for conformal X-band MIMO applications, making it an excellent choice for such use cases.

## Data Availability

All data generated or analysed during this study are included in this published article.
